# Cardiopulmonary exercise testing in patients with moderate-severe obesity: a clinical evaluation tool for OSA?

**DOI:** 10.1007/s11325-021-02475-0

**Published:** 2021-09-06

**Authors:** Marco Vecchiato, Daniel Neunhaeuserer, Giulia Quinto, Silvia Bettini, Andrea Gasperetti, Francesca Battista, Andrea Vianello, Roberto Vettor, Luca Busetto, Andrea Ermolao

**Affiliations:** 1 Sports and Exercise Medicine Division, Department of Medicine, University of Padova, Via Giustiniani 2, 35128 Padova, Italy; 2 Clinical Network of Sport and Exercise Medicine of the Veneto Region, Padova, Italy; 3 Center for the Study and Integrated Treatment of Obesity (CeSTIO), Internal Medicine 3, Department of Medicine, University of Padova, Padova, Veneto Region Italy; 4 Respiratory Pathophysiology Unit, Department of Cardiological, Thoracic and Vascular Sciences, University of Padova, Padova, Italy

**Keywords:** Obstructive sleep apnea, Cardiorespiratory fitness, Cardiorespiratory sleep study, Continuous positive airway pressure, End-tidal carbon dioxide

## Abstract

**Purpose:**

Obstructive sleep apnea (OSA) is a widespread comorbidity of obesity. Nasal continuous positive airway pressure (CPAP) has been demonstrated very effective in treating patients with OSA. The aims of this study were to investigate whether or not cardiopulmonary exercise testing (CPET) can characterize patients with OSA and to evaluate the effect of nasal CPAP therapy.

**Methods:**

An observational study was conducted on patients with moderate to severe obesity and suspected OSA. All patients underwent cardiorespiratory sleep study, spirometry, and functional evaluation with ECG-monitored, incremental, maximal CPET.

**Results:**

Of the 147 patients, 94 presented with an apnea–hypopnea index (AHI) ≥ 15 events/h and were thus considered to have OSA (52 receiving nasal CPAP treatment; 42 untreated) while 53 formed a control group (AHI < 15 events/h). Patients with untreated OSA showed significantly lower oxygen uptake (VO_2_), heart rate, minute ventilation (VE), and end tidal carbon dioxide (PETCO_2_) at peak exercise compared to controls. Patients receiving nasal CPAP showed higher VE and VO_2_ at peak exercise compared to untreated patients. A difference in PETCO_2_ between the maximum value reached during test and peak exercise (ΔPETCO_2_ max-peak) of 1.71 mmHg was identified as a predictor of OSA.

**Conclusion:**

Patients with moderate to severe obesity and untreated OSA presented a distinctive CPET-pattern characterized by lower aerobic and exercise capacity, higher PETCO_2_ at peak exercise associated with a lower ventilatory response. Nasal CPAP treatment was shown to positively affect these cardiorespiratory adaptations during exercise. ΔPETCO_2_ max-peak may be used to suggest OSA in patients with obesity.

## Introduction

Obstructive sleep apnea (OSA) is a relatively common condition in the adult population, closely associated with obesity [1]. OSA is characterized by repeated episodes of apnea and hypopnea during sleep resulting in intermittent hypoxia and hypercapnia, frequent arousals with sleep fragmentation, and daytime sleepiness. It also leads to secondary sympathetic activation, oxidative stress, systemic inflammation, and increased cardiovascular risk [2].

Cardiopulmonary exercise testing (CPET) is highly recommended for patients with increased cardiovascular risk and chronic diseases, like obesity. Indeed, different CPET parameters have been proposed as important prognostic markers, while others are used for diagnostic testing and cardiopulmonary screening. Despite an increasing scientific interest in OSA in recent years [3], very few data investigating the effect of OSA on cardiopulmonary function in patients with severe obesity are currently available [4, 5]. Most studies have shown reduced maximal aerobic exercise capacity in patients with OSA compared to control subjects [6, 7]. Previous studies showed improvements in cardiac function and cardiorespiratory fitness in patients with OSA after eight weeks of nasal continuous positive airway pressure (CPAP) treatment [8, 9]. Indeed, nasal CPAP is the gold-standard treatment for moderate to severe OSA and has been proven to reduce cardiovascular mortality and non-fatal cardiovascular events in this population [10].

The present study aimed, first to characterize the cardiopulmonary function of patients with moderate to severe obesity affected by OSA, investigating a possible distinctive cardiopulmonary response to exercise. Second, we aimed to evaluate if CPAP therapy may affect cardiorespiratory efficiency and ventilatory drive during exercise.

## Material and methods

### Participants and protocol

In this observational cross-sectional study, we evaluated 185 patients affected by moderate to severe obesity and suspected OSA, consecutively recruited within the Veneto Region diagnostic-therapeutic pathway for patients with obesity. These patients underwent cardiorespiratory sleep study analysis at University Hospital of Padova from February 2014 to January 2020 for suspicion of OSA based on clinical evaluation and a validated questionnaire (Epworth Sleepiness Scale). During the same period, these patients had been referred for functional evaluation to the Sport and Exercise Medicine Division. This study was performed in accordance with the Declaration of Helsinki and approved by the local ethics committee (99n/AO/21); all participants provided written informed consent. Inclusion criteria were age between 18 and 70 years, body mass index (BMI) > 35 kg/m^2^, and suspected OSA. Exclusion criteria were significant heart, lung, or musculoskeletal disease that would impede maximal exercise testing. A subgroup of patients affected by OSA was treated with CPAP for at least 8 weeks before functional evaluation. The remaining patients with OSA were not receiving CPAP due to intolerance or were waiting to start therapy. Thus, the patients were divided in three groups:

- Patients affected by obesity (Ob)
- Patients affected by obesity and OSA (Ob-OSA)
- Patients affected by obesity and OSA treated with CPAP (Ob-CPAP)

### Cardiorespiratory sleep study

The cardiorespiratory sleep study was performed using the SOMNOtouchTM® NIBP device (SOMNOmedics Italia), for at least 8 h per night. Peripheral oxygen saturation, respiratory flow in the upper airways, respiratory movements of the chest and abdomen, snoring phases, sleeping position, blood pressure levels, and ECG were recorded. These data were automatically analyzed by the DOMINO software® and subsequently validated by an expert operator according to the most recent American Academy of Sleep Medicine Reviewer criteria [11]. The polysomnographic data encompassed Apnea Hypopnea Index (AHI), minimum percentage oxyhemoglobin saturation (mSaO2%), mean percentage oxyhemoglobin saturation (meanSaO2%), percentage of time with oxyhemoglobin saturation percentage less than 90 (TS < 90%), and number of desaturations with oxyhemoglobin saturation < 90% (nSaO2 < 90%). We were unable to detect peripheral oxygen saturation of 13 participants. In this study, OSA was considered to be present in patients with an AHI of 15 or more events/h [11].

### Cardiopulmonary exercise testing

Each patient was subsequently evaluated with incremental, maximal, ECG-monitored CPET (Jaeger Masterscreen-CPX, Carefusion). All tests were performed on treadmill (T170 DE, Cosmed), using the modified Bruce protocol. Criteria of exhaustion were a Borg rating of perceived exertion ≥ 18/20 associated with a Respiratory Exchange Ratio (RER) > 1.10, and/or a peak heart rate (HR) ≥ 85% of predicted HR max, and/or the achievement of a plateau of oxygen uptake (VO_2_). Arterial blood pressure and peripheral oxygen saturation were continuously monitored. Ventilatory and gas exchange measurements were sampled breath-by-breath to assess: VO_2_, minute ventilation (VE), ventilatory equivalents for carbon dioxide (VE/VCO_2_) and end-tidal pressures for oxygen and carbon dioxide (PETO_2_ and PETCO_2_). VE, VO_2_ and PETCO_2_ were determined also at the anaerobic threshold (AT) and respiratory compensation point (RCP) [12]. Differences between two PETCO_2_ values were presented as ΔPETCO_2_, i.e., the difference between the maximum PETCO_2_ value reached during testing and PETCO_2_ at peak exercise was defined as ΔPETCO_2_ max-peak.

### Statistical analysis

Statistical analyses were performed with Statistical Package for Social Science (SPSS Inc., Chicago). Continuous variables are expressed as mean ± standard deviation and comparison between subgroups was performed with the one-way analysis of variance test with Bonferroni correction. Categorical variables were compared between groups using Pearson’s chi squared test. The relationship between continuous variables was evaluated by Spearman’s correlation coefficient (r). Receiver operating characteristic (ROC) curve was constructed to identify a parameter that can be used to discriminate Ob-OSA from Ob, maximizing sensitivity and specificity values. All reported probability values were two-tailed and a value of *p* < 0.05 was considered statistically significant.

## Results

**Fig. 1:**
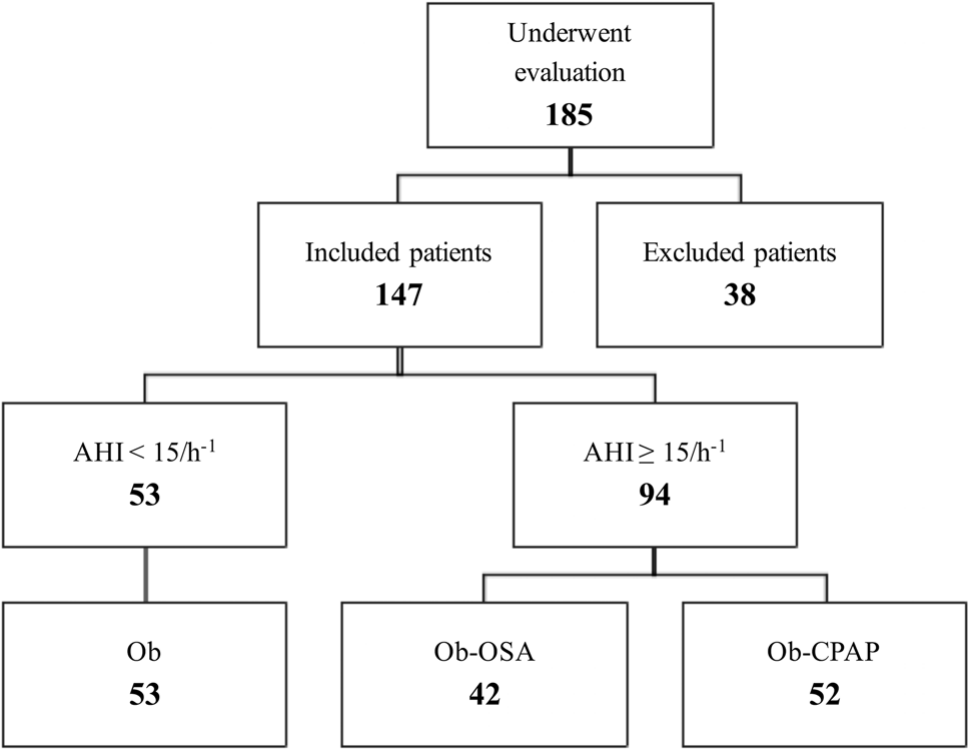
Study flow-chart. AHI, apnea–hypopnea index; Ob, patients affected by obesity; Ob-OSA, patients affected by obesity and OSA; Ob-CPAP, patients affected by obesity and OSA treated with CPAP

Of 147 patients with moderate to severe obesity eligible for study inclusion, 94 patients with OSA (52 Ob-CPAP and 42 Ob-OSA) were compared with 53 patients without OSA (Ob; Fig. 1). Demographic and anthropometric characteristics, lung function, and cardiorespiratory sleep study measurements of patients are shown in Table 1. No differences in age, gender, BMI, or sedentary lifestyle were found between groups. Major co-morbidities were dyslipidemia (68%), arterial hypertension (52%), and diabetes mellitus (23%); 30 patients were active smokers (20%).

**Table 1:** Baseline clinical characteristics, simple lung spirometry, and cardiorespiratory sleep study measurements of the study population (*n* = 147)

	Controls [Ob] (*n* = 53)	Untreated [Ob-OSA] (*n* = 42)	Treated [Ob-CPAP] (*n* = 52)	*p*
Gender (male %)	34 (65)	25 (60)	38 (73)	.363
Age (years)	48.0 ± 10.8	51.2 ± 12.6	51.3 ± 9.4	.220
BMI (kg/m^2^)	44.4 ± 6.0	46.6 ± 8.2	45.2 ± 6.0	.286
Hypertension (%)	22 (42)	23 (55)	32 (62)	.113
Diabetes (%)	14 (26)	6 (14)	14 (27)	.274
Smoking (%)	9 (17)	11 (26)	10 (19)	.814
Sedentary (%)	42 (79)	31 (74)	40 (77)	.823
FEV1 (%)	108.67 ± 18.27	98.37 ± 16.63	99.18 ± 15.77	.003*
FVC (%)	104.31 ± 17.92	93.00 ± 14.74	96.04 ± 15.69	.004*
FEV1/FVC (%)	78.96 ± 12.63	76.55 ± 14.11	77.66 ± 12.46	.666
PEF (%)	99.46 ± 18.25	93.37 ± 20.33	99.00 ± 17.65	.236
AHI (events/h)	5.4 ± 4.0	34.0 ± 16.8	38.6 ± 16.9	< .001*§
minSaO_2_ (%)	85.22 ± 5.84	76.33 ± 10.13	78.15 ± 7.28	< .001*§
meanSaO_2_ (%)	94.84 ± 1.54	93.44 ± 2.48	93.55 ± 1.70	.001*§
TS < 90% (%)	2.84 ± 5.55	13.61 ± 16.47	15.46 ± 23.12	< .001*§
nSaO2 < 90%	36.20 ± 9.81	104.22 ± 23.97	109.66 ± 17.75	.002*§

Continuous variables are expressed as mean ± standard deviation and categorical variables are expressed as frequencies (percentage). *AHI*, apnea–hypopnea index; *BMI*, body mass index; *FEV1*, forced expiratory volume in 1^st^ s; *FVC*, forced vital capacity; *minSaO*_*2*_, minimum percentage oxyhemoglobin saturation; *meanSaO*_*2*_, mean percentage oxyhemoglobin saturation; *nSaO*_*2*_ < *90%*, number of desaturations with oxyhemoglobin saturation < 90%; *PEF*, peak expiratory force; *TS* < *90%*, percentage of time with oxyhemoglobin saturation percentage less than 90%

^*^ *p* < .05 between Ob-OSA and Ob. ‡ p < .05 between Ob-OSA and Ob-CPAP. § p < .05 between Ob-CPAP and Ob

Resting and exercise CPET parameters are presented in Table 2. Ob-OSA presented lower VO_2_ peak/kg when compared to Ob and Ob-CPAP (*p* = 0.014 and *p* = 0.019, respectively). Indeed, not considering patients in CPAP treatment, a significant, although weak, inverse correlation between VO_2_ peak/kg and AHI was identified (r =  − 0.242, *p* = 0.018).

**Table 2:** Cardiopulmonary test parameters of the study population (*n* = 147)

	Controls [Ob] (*n* = 53)	Untreated [Ob-OSA] (*n* = 42)	Treated [Ob-CPAP] (*n* = 52)	*p*
Rest parameters				
SaO_2_ rest (%)	99.47 ± 0.67	98.98 ± 0.92	98.88 ± 1.67	.027*§
HR rest (bpm)	78.77 ± 11.84	79.98 ± 15.54	77.19 ± 10.71	.564
SBP rest (mmHg)	127.45 ± 16.80	125.55 ± 17.11	128.38 ± 13.25	.684
DBP rest (mmHg)	76.70 ± 10.69	80.12 ± 9.33	77.33 ± 11.28	.212
BF rest (min^−1^)	18.89 ± 5.38	20.00 ± 5.35	20.08 ± 5.71	.472
VE rest (L/min)	14.51 ± 5.60	15.61 ± 4.88	16.31 ± 4.88	.259
Exercise parameters				
SaO_2_ peak (%)	97.92 ± 1.07	97.64 ± 1.67	97.21 ± 2.41	.139
VO_2_ peak (mL)	2546.45 ± 648.07	2455.36 ± 619.40	2717.98 ± 559.53	.105
VO_2_ peak (mL/Kg/min)	20.12 ± 3.56	17.99 ± 4.00	20.05 ± 3.25	.007*‡
VO_2_ peak (% of predicted)	108.81 ± 15.12	98.64 ± 20.24	103.73 ± 18.79	.026*
VO_2_ AT (%)	67.42 ± 9.50	69.91 ± 9.52	67.48 ± 8.18	.334
VO_2_ RCP (%)	82.55 ± 8.66	85.71 ± 6.53	82.88 ± 11.74	.216
OUES (ml/logL)	2633.79 ± 671.78	2706.00 ± 735.85	2934.69 ± 848.24	.111
Exercise time (sec)	994.04 ± 180.16	886.98 ± 200.88	937.44 ± 205.13	.031*
HR peak (bpm)	158.42 ± 15.78	146.93 ± 19.08	150.35 ± 14.88	.003*
HR peak (% of predicted)	90.40 ± 7.45	85.21 ± 9.52	87.48 ± 8.97	**.**015*
HR reserve (bpm)	80.17 ± 19.49	66.86 ± 21.24	73.60 ± 16.47	**.**004*
HR recovery (bpm)	19.17 ± 9.15	16.67 ± 8.23	19.02 ± 8.76	.317
SBP peak (mmHg)	172.57 ± 27.20	166.62 ± 25.51	171.96 ± 22.75	.469
DBP peak (mmHg)	76.89 ± 14.91	85.69 ± 10.79	77.81 ± 13.94	**.**002*‡
O_2_ pulse (ml/bpm)	16.15 ± 3.62	17.27 ± 4.35	17.91 ± 3.73	.066
RER peak	1.14 ± 0.07	1.10 ± 0.09	1.16 ± 0.08	**.**004‡
VE/VCO_2_ slope	26.09 ± 3.69	25.89 ± 3.95	25.73 ± 3.19	.880
VE peak (L/min)	81.58 ± 22.24	75.19 ± 17.76	89.50 ± 19.01	**.**003‡§
BR (%)	23.49 ± 14.33	26.43 ± 15.28	20.48 ± 13.98	.143
BF peak (min^−1^)	39.26 ± 7.73	37.00 ± 6.58	38.35 ± 6.73	.303

Data are expressed as mean ± standard deviation. *AT*, anaerobic threshold; *BR*, breathing reserve; *BF*, breathing frequency; *DBP*, diastolic blood pressure; *HR*, heart rate; *O*_*2*_* pulse*, ratio between oxygen uptake and heart rate; *OUES*, oxygen uptake efficiency slope; *RCP*, respiratory compensation point; *RER*, respiratory exchange ratio; *SBP*, systolic blood pressure; *SaO*_*2*_, blood oxygen saturation with pulse oximetry; *VE*, minute ventilation; *VE/VCO*_*2*_, minute ventilation/carbon dioxide production slope; *VO*_*2*_, oxygen uptake

^*^ *p* < .05 between Ob-OSA and Ob. ‡ *p* < .05 between Ob-OSA and Ob-CPAP. § *p* < .05 between Ob-CPAP and Ob

Evaluating patients’ cardiovascular response to exercise, data show that Ob-OSA presented increased diastolic blood pressure (DBP) at peak exercise compared to Ob-CPAP and Ob (*p* = 0.015 and *p* = 0.003, respectively), while no significant difference between groups on systolic blood pressure (SBP) at rest and at peak exercise was observed. Furthermore, maximal HR and HR reserve were higher in Ob than in Ob-OSA (both *p* = 0.003).

**Fig. 2:**
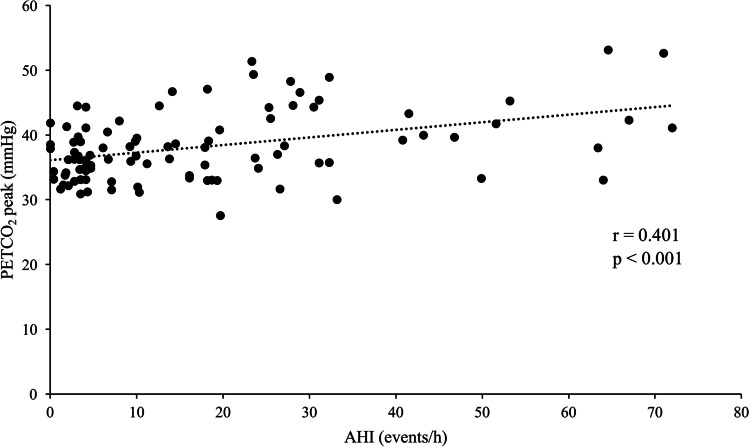
Relationship between PETCO_2_ peak and AHI. Figure 2 shows the positive correlation between end tidal carbon dioxide at peak exercise (PETCO_2_ peak) and apnea–hypopnea index (AHI)

**Fig. 3:**
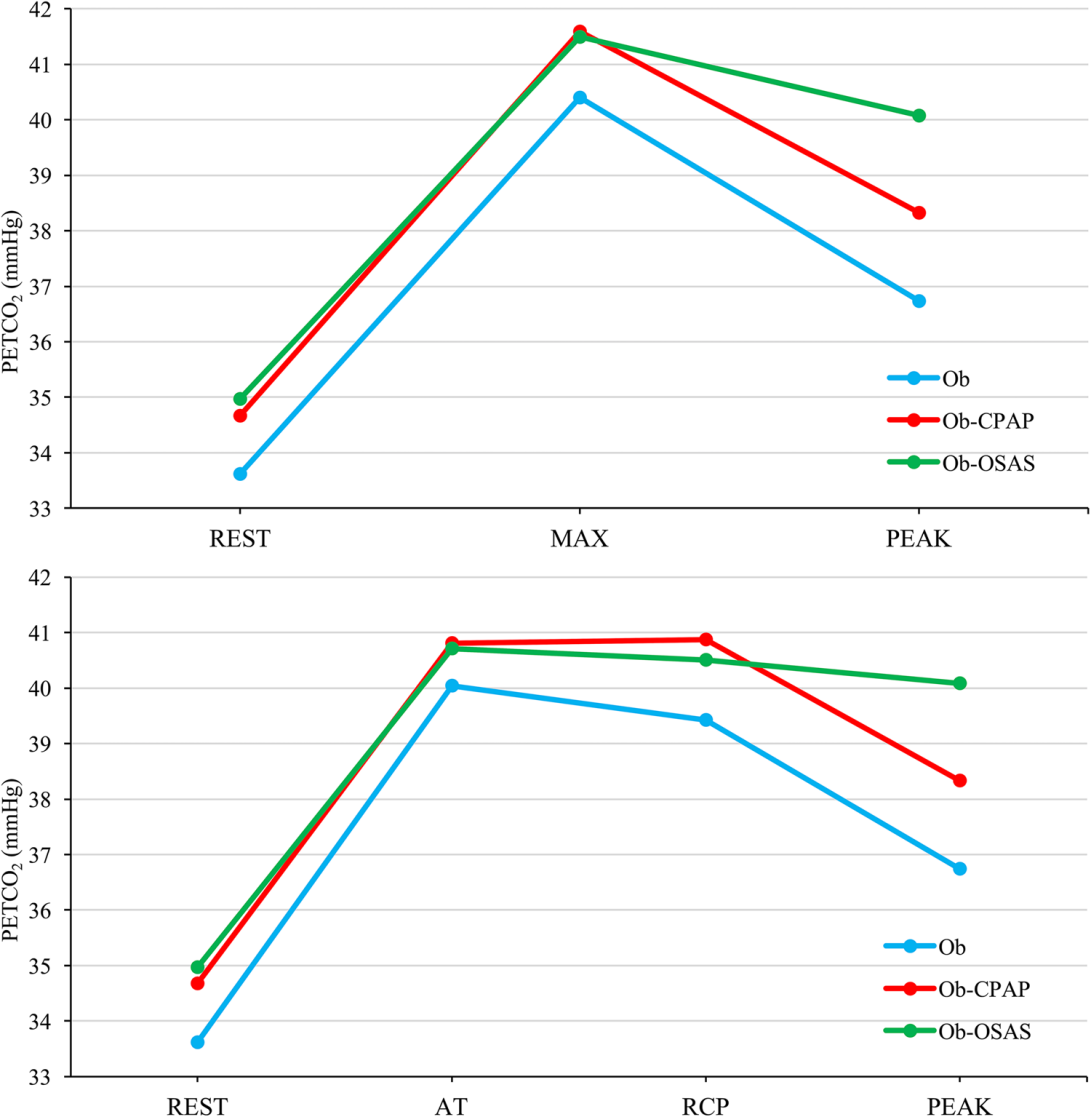
Response of PETCO_2_ during incremental exercise. Figure 3 shows the response of end tidal carbon dioxide (PETCO_2_) during incremental cardiopulmonary exercise testing in Ob (blue), Ob-CPAP (red), and Ob-OSA (green). Although PETCO_2_ max is similar among the three groups, it diverges at peak exercise (graphic above). In the graphic, it is possible notice that these differences in PETCO_2_ peak occur after RCP is achieved. REST, at rest; AT, anaerobic threshold; RCP, respiratory compensation point; PEAK, at peak exercise; MAX, maximum value reached during exercise testing

**Fig. 4:**
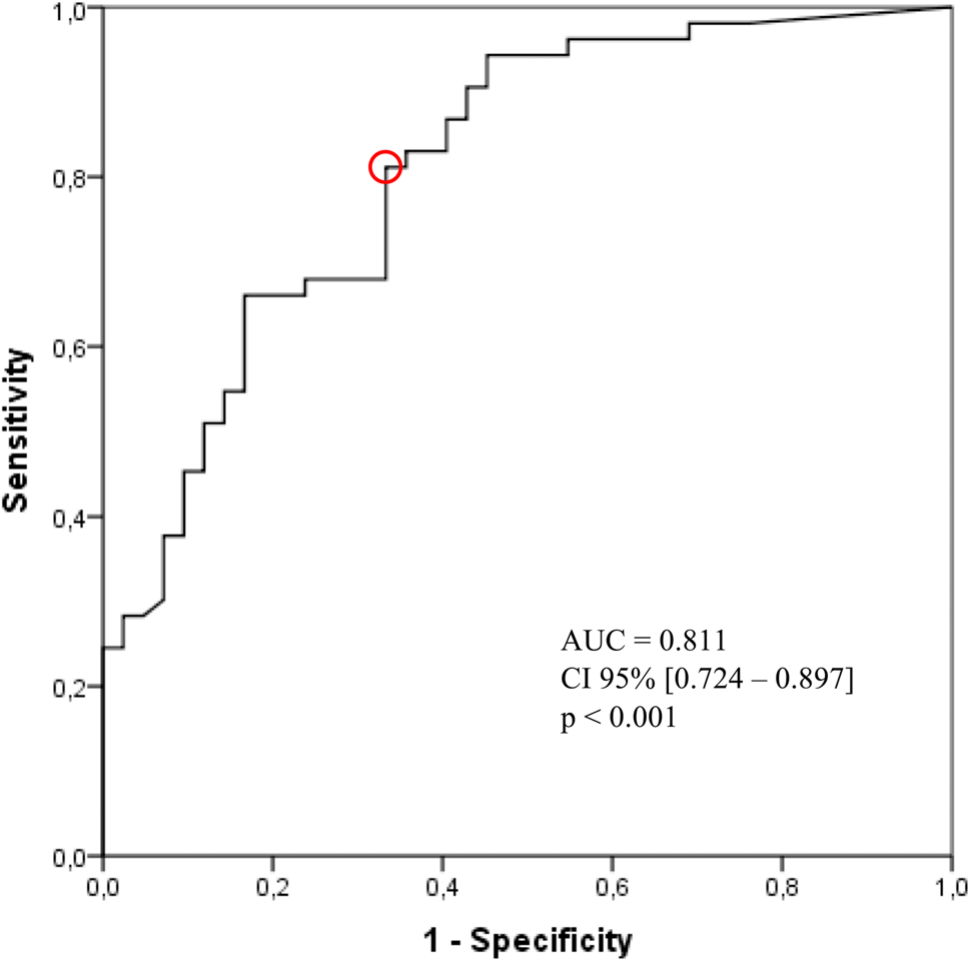
ROC curve analysis of ΔPETCO_2_ max-peak. Figure 4 shows receiver operating characteristic curve analysis of the difference in end tidal carbon dioxide pressure between the maximum value reached during exercise and peak exercise (ΔPETCO_2_ max-peak), as predictor of OSA. The red circle indicates the value of 1.71 mmHg, having a sensitivity of 81% and a specificity of 67%. AUC, area under the curve; CI 95%, 95% confidence interval

Finally, regarding patients’ respiratory gas exchange, Ob-OSA presented lower maximal VE compared to Ob-CPAP (*p* = 0.002) and higher PETCO_2_ at peak exercise (PETCO_2_ peak) compared to Ob (*p* = 0.004; Table 3). Indeed, not considering patients in CPAP treatment, PETCO_2_ peak showed a statistically significant positive correlation with AHI (r = 0.401, *p* < 0.001; Fig. 2). Moreover, data revealed a lower ΔPETCO_2_ max-peak when Ob-OSA was compared with Ob-CPAP and Ob (both *p* < 0.001; Table 3 and Fig. 3). ROC analysis demonstrated that ΔPETCO_2_ max-peak, used as predictor of Ob-OSA, showed an area under the curve of 0.811. A cut-off value of 1.71 mmHg can be proposed with a sensitivity of 81% and a specificity of 67% (Fig. 4).

**Table 3:** End tidal carbon dioxide pressure (PETCO_2_) of the study population (*n* = 147) measured during cardiopulmonary exercise testing

	Controls [Ob] (*n* = 53)	Untreated [Ob-OSA] (*n* = 42)	Treated [Ob-CPAP] (*n* = 52)	*p*
PETCO_2_ rest	33.62 ± 3.27	34.97 ± 5.04	34.67 ± 3.42	.201
PETCO_2_ AT	40.05 ± 4.22	40.71 ± 5.70	40.81 ± 3.94	.661
PETCO_2_ RCP	39.42 ± 4.04	40.51 ± 6.05	40.87 ± 4.18	.274
PETCO_2_ peak	36.74 ± 3.82	40.08 ± 6.44	38.33 ± 4.48	.005*
PETCO_2_ max	40.40 ± 4.07	41.49 ± 6.00	41.59 ± 4.14	.372
ΔPETCO_2_ rest-AT	-5.64 ± 6.88	-5.74 ± 3.30	-6.71 ± 5.20	.550
ΔPETCO_2_ AT-RCP	0.63 ± 1.63	0.20 ± 1.45	-0.06 ± 1.99	.125
ΔPETCO_2_ RCP-peak	2.73 ± 1.97	0.43 ± 1.73	2.53 ± 1.83	** < .**001*‡
ΔPETCO_2_ max-peak	3.74 ± 2.37	1.41 ± 1.43	3.26 ± 2.05	** < .**001*‡

All data are expressed in mmHg and represented as mean ± standard deviation. *AT*, anaerobic threshold; *PETCO*_*2*_, end tidal carbon dioxide pressure; *RCP*, respiratory compensation point; *ΔPETCO*_*2*_, difference in value of end tidal carbon dioxide pressure between two distinct points during cardiopulmonary exercise test (i.e., ΔPETCO_2_ max-peak is the difference in PETCO_2_ between the maximum value reached during exercise and peak exercise)

^*^ *p* < .05 between Ob-OSA and Ob. ‡ *p* < .05 between Ob-OSA and Ob-CPAP. § *p* < .05 between Ob-CPAP and Ob

## Discussion

This is the largest single study reporting cardiopulmonary exercise parameters in patients with OSA and moderate to severe obesity. To date, CPET has not been considered a useful tool in the evaluation of patients with suspected OSA, although the literature has already described distinguishing features that might be useful in the diagnostic process as well as during follow-up. This work aims to provide further evidence for the potential utility of CPET in the clinical management of these patients. The main results of our study are the following:

1. Patients with moderate to severe obesity and OSA showed reduced aerobic capacity and exercise tolerance compared to patients with moderate to severe obesity without OSA.
2. Although patients with OSA showed higher PETCO_2_ at peak exercise, a reduced ventilatory drive was observed.
3. ΔPETCO_2_ max-peak may be proposed as a marker in CPET for patients with obesity and suspected OSA.
4. Patients with OSA receiving CPAP therapy showed a cardiorespiratory response to exercise similar to controls without OSA.

### Cardiorespiratory fitness

According to our data, Ob-OSA presented with lower cardiorespiratory fitness compared to Ob and Ob-CPAP. Different previous studies have evaluated exercise capacity in patients with OSA but only two of them analyzed patients with severe obesity [4, 5]. A recent systematic review and meta-analysis showed a reduced maximal aerobic capacity in patients with OSA, confirming its significant impact on patients’ cardiorespiratory fitness [3]. The reasons for these exercise limitations are not fully understood but different explanations have been proposed, suggesting a multifactorial impairment.

Patients with OSA present more frequently left and right ventricular diastolic dysfunction, respiratory alterations, restrictive pulmonary disease, and pulmonary hypertension, which may all contribute to a reduced maximal aerobic capacity [13, 14]. For this reason, patients affected by these diseases were excluded from this study. Indeed, lung function tests, breathing reserve, oxygen pulse at peak, and the absence of peripheral desaturation during exercise with normal VE/VCO_2_ slope were not consistent with major pulmonary or cardiac limitations to exercise.

Furthermore, a decreased maximal lactate concentration and its delayed elimination has been observed in patients with OSA during exercise when compared to age and BMI matched controls and this may indicate impaired glycolytic metabolism and reduced exercise tolerance [15].

Also, musculoskeletal damage has been proposed as possible cause of exercise impairment. Indeed, muscular biopsies have demonstrated structural and bio-energetic changes in skeletal muscle fibers, probably due to continuous or intermittent hypoxia [16].

Sleep-related hypopnea leads to excessive daytime sleepiness, which may affect the ability to achieve maximum exercise workload. Sleep deprivation has already been identified as a limiting factor in exercise time because it seems to increase perceived maximal effort [17]. Moreover, aerobic capacity is influenced by physical activity level, which is known to be lower in patients with OSA [18, 19].

Ob-CPAP exhibited higher aerobic capacity than Ob-OSA, comparable with controls. The continuous CPAP treatment effect on aerobic capacity in patients with OSA has been already described [2]. In fact, CPAP therapy for at least eight consecutive weeks was predominantly associated with significant improvements in VO_2_ max [8, 9, 20, 21]. CPAP therapy may normalize gas exchanges during sleep and contribute to structural and bio-energetic changes in skeletal muscles. Furthermore, CPAP associated reduction of sleep deprivation and better daytime alertness may help to increase motivation and performance during CPET, consequently leading to higher maximal aerobic capacity. Indeed, though CPAP therapy does not seem to improve quality of life scores, it has been shown to increase physical domains and vitality of patients with OSA patients [22].

### Cardiovascular response

The reduced aerobic capacity found in Ob-OSA may at least in part be due to a lower HR response during exercise [4, 23]. Indeed, several other studies have reported a chronotropic impairment at peak exercise in patients with OSA suggesting a downregulation of beta-adrenergic receptors consequent to sympathetic hyperactivity [24].

The cardiovascular response to exercise was also characterized by a higher DBP at peak exercise in Ob-OSA, which is in line with results of previous studies [15, 25]. However, the Ob-CPAP group showed a similar DBP response during exercise compared to Ob. Indeed, CPAP therapy has been associated with a decrease in sympathetic hyperactivity as assessed by HR variability [20]. Excessive sympathetic activity may contribute to limit maximal aerobic capacity through peripheral vasoconstriction probably due to the activation of excitatory chemoreflex afferents.

### Ventilatory response and gas-exchange

OSA is characterized by recurrent upper airway collapse during sleep which may cause CO_2_ retention leading to the onset of respiratory acidosis, resulting in compensatory renal retention of bicarbonate ions. This condition leads to a subsequent reduced respiratory frequency and daily ventilation. Although resting ventilation was similar between the three study groups, data showed a reduced ventilatory response to exercise in Ob-OSA. The continuous stimulus by chronic CO_2_ retention might cause a dysregulation of the metabolic set point that affects the ventilatory drive, causing it to be less sensitive to CO_2_ levels [26]. Moreover, a reduced ventilatory response at high exercise intensities may reduce patients’ exercise tolerance due to limited metabolic buffering and directly influence their maximal aerobic capacity. Indeed, the blunted respiratory drive cannot compensate for the increased respiratory demand during exercise and thus patients with OSA cannot eliminate the extra amount of CO_2_ produced during exercise, causing increased levels of PETCO_2_ at elevated intensities [27].

PETCO_2_ presents a specific pattern during exercise, which is mainly influenced by progressive accumulation of lactic acid during incremental exercise, the subsequent metabolic buffering, and the associated ventilatory response. PETCO_2_ rises until reaching the AT, remaining constant during isocapnic buffering until the RCP is reached. Afterwards, a further ventilatory surge exceeds the increase in the respiratory elimination of CO_2_ causing a physiological reduction in PETCO_2_. The trend of PETCO_2_ during the exercise phases seems similar in the different study groups until reaching the RCP, when Ob-OSA showed a lower decreasing trend in PETCO_2_ up to peak exercise. Indeed, 16 patients presented a PETCO_2_ peak higher than PETCO_2_ at the RCP and 15 of them belonged to Ob-OSA group. This is further supported by the positive correlation between PETCO_2_ peak and the severity of OSA. Despite the role of PETCO_2_ has already been evaluated during sleep in patients with OSA [28], there are only few published data investigating its behavior during exercise, and no information is currently available during CPET in patients with moderate-severe obesity. Our study outcomes in this specific population are in line with preceding studies showing higher PETCO_2_ at peak exercise, while these alterations were positively affected when patients were treated with CPAP [8, 29, 30].

ΔPETCO_2_ max-peak is an objective and reproducible index that is unaffected by influences related to threshold determination and it can be easily measured in any CPET. ROC analysis showed that ΔPETCO_2_ max-peak was a good predictor of OSA and a cut-off value of 1.71 mmHg can be proposed with a good sensitivity and a fair specificity. However, a reduction of PETCO_2_ of less than 2 mmHg at peak exercise might be of interest for clinical decision making (sensitivity 74%, specificity 67%). Considering that OSA is more frequent in patients with moderate-severe obesity, this cut-off may help physicians to better interpret CPET values according to patients’ history and symptoms, and to further investigate the potential presence of apnea/hypopnea during sleep, when appropriate.

### Limitations and perspectives

In this study, patients’ physical activity level was not recorded. Thus, it could not be excluded that differences in VO_2_ peak between Ob-OSA and other groups may be due, at least in part, to training levels. Indeed, a proposal for future studies could be the implementation of an objective physical activity monitoring system via accelerometers or at least via a physical activity screening questionnaire.

PETCO_2_ has been used to indirectly estimate arterial CO_2_ pressure but these values may not match because of ventilation-perfusion mismatch. However, the exclusion criteria used in this study and the substantial normality of VE/VCO_2_ slope values should have minimized the risk of such pathological conditions. Future research projects may provide arterial or transcutaneous blood gases measurements to further address these issues.

## Conclusion

CPET is a safe and non-invasive evaluation for all patients with chronic diseases, including obesity. Patients with moderate to severe obesity and OSA presented reduced aerobic capacity, exercise tolerance, and ventilatory response with an associated higher PETCO_2_ at peak exercise. We suggest ΔPETCO_2_ max-peak as an objective and easily reproducible predictor of OSA. Therefore, outpatient screening with CPET may also provide useful information for the early identification of patients with suspected OSA. Finally, CPET may also be useful for the follow-up of patients with OSA and the evaluation of the effect of CPAP therapy.

## Data Availability

The data that support the findings of this study are available from the corresponding author upon reasonable request.

## Abbreviations

AHI: Apnea–hypopnea index
AT: Anaerobic threshold
BMI: Body mass index
CPAP: Continuous positive airway pressure
CPET: Cardio-pulmonary exercise testing
DBP: Diastolic blood pressure
FEV1: Forced expiratory volume in 1^st^ s
FVC: Forced vital capacity
HR: Heart rate
O_2_ pulse: Ratio between oxygen uptake and heart rate
OSA: Obstructive sleep apnea syndrome
PEF: Peak expiratory flow
PETCO_2_: End-tidal carbon dioxide pressure
RCP: Respiratory compensation point
RER: Respiratory exchange ratio
SBP: Systolic blood pressure
SpO_2_: Blood oxygen saturation with pulse oximetry
VE: Minute ventilation
VE/VCO_2_: Minute ventilation/carbon dioxide production slope
VO_2_: Oxygen uptake
